# Drug repurposing-based nanoplatform via modulating autophagy to enhance chemo-phototherapy against colorectal cancer

**DOI:** 10.1186/s12951-024-02416-5

**Published:** 2024-04-24

**Authors:** Ke Ding, Hailong Tian, Lei Li, Zhihan Wang, Shanshan Liu, Ning Ding, Edouard C. Nice, Canhua Huang, Jinku Bao, Wei Gao, Zheng Shi

**Affiliations:** 1 Clinical Medical CollegeAffiliated Hospital of Chengdu University, Chengdu University, Chengdu, 610106 China; 2 Department of Clinical Pharmacy, School of Pharmacy, Zunyi Medical University, Zunyi, 563006 China; 3 State Key Laboratory of Biotherapy and Cancer Center, West China Hospital, and West China, School of Basic Medical Sciences & Forensic Medicine, Collaborative Innovation Center for Biotherapy, Sichuan University, Chengdu, 610041 China; 4 Department of anorectal surgery, Hospital of Chengdu University of Traditional Chinese Medicine and Chengdu University of Traditional Chinese Medicine, Chengdu, 610072 China; 5 Shanghai municipal Hospital of Traditional Chinese Medicine, Shanghai University of Traditional Chinese Medicine, Shanghai, 201203 China; 6 Department of Biochemistry and Molecular Biology, Monash University, Clayton, VIC 3800 Australia

**Keywords:** Colorectal cancer, Autophagy, Drug repurposing, Chemo-phototherapy

## Abstract

Multi-modal combination therapy is regarded as a promising approach to cancer treatment. Combining chemotherapy and phototherapy is an essential multi-modal combination therapy endeavor. Ivermectin (IVM) is a potent antiparasitic agent identified as having potential antitumor properties. However, the fact that it induces protective autophagy while killing tumor cells poses a challenge to its further application. IR780 iodide (IR780) is a near-infrared (NIR) dye with outstanding photothermal therapy (PTT) and photodynamic therapy (PDT) effects. However, the hydrophobicity, instability, and low tumor uptake of IR780 limit its clinical applications. Here, we have structurally modified IR780 with hydroxychloroquine, an autophagy inhibitor, to synthesize a novel compound H780. H780 and IVM can form H780-IVM nanoparticles (H-I NPs) via self-assembly. Using hyaluronic acid (HA) to modify the H-I NPs, a novel nano-delivery system HA/H780-IVM nanoparticles (HA/H-I NPs) was synthesized for chemotherapy-phototherapy of colorectal cancer (CRC). Under NIR laser irradiation, HA/H-I NPs effectively overcame the limitations of IR780 and IVM and exhibited potent cytotoxicity. In vitro and in vivo experiment results showed that HA/H-I NPs exhibited excellent anti-CRC effects. Therefore, our study provides a novel strategy for CRC treatment that could enhance chemo-phototherapy by modulating autophagy.

**Supplementary Information:**

The online version contains supplementary material available at 10.1186/s12951-024-02416-5.

## Introduction

Colorectal cancer (CRC) is the third most common malignant tumor worldwide. Current evidence estimates that there are approximately 1.85 million new cases and 850,000 fatalities yearly [1–3]. Despite advances in colorectal cancer treatment regimens, conventional chemotherapy remains an indispensable tool in CRC therapy. However, the complex tumor microenvironment of CRC and side effects caused by nontargeted chemotherapeutic drugs greatly impair the efficacy of conventional chemotherapy, thereby increasing the burden on patients [4].

Ivermectin (IVM), a macrolide compound, is widely used for antiparasitic therapy [5]. Due to its outstanding safety and reliability in clinical use over many years, IVM has emerged as a promising cancer treatment candidate [6]. Recent studies have shown that IVM acts as a broad-spectrum antitumor drug, which can kill tumor cells and cause cytoprotective autophagy against cell death [7]. Combining IVM with an autophagy inhibitor has been shown to enhance its tumor-killing effect [8, 9]. However, the limited water solubility and poor bioavailability of IVM have hampered its clinical application. Therefore, it is of utmost importance to resolve the issue of poor water solubility and low bioavailability of IVM, as well as its ability to induce protective autophagy in tumor cells.

In clinical practice, hydroxychloroquine (HCQ) is a commonly used antimalarial and antirheumatic agent [10]. Studies have shown that HCQ has the potential to inhibit tumor growth through multiple mechanisms, with autophagic flux inhibition being the most widely accepted mechanism [11, 12]. In addition, numerous studies have demonstrated that the co-administration of HCQ with other pharmacological drugs that induce cytoprotective autophagy can bolster the chemotherapeutic efficacy against cancers [13–15]. Using HCQ as an adjuvant to improve the therapeutic efficacy of chemotherapeutic drugs is therefore a promising strategy.

Phototherapy is an innovative approach for treating tumors, which utilizes photosensitizers to convert light energy into chemical and thermal energy, damaging macromolecules in cells, leading to acute cell injury and, ultimately, tumor cell death [16–18]. In recent years, phototherapy has gained widespread interest due to its low toxicity, high spatial selectivity, non-invasiveness, and negligible drug resistance compared to other traditional treatment options [19, 20]. IR780 is a photosensitizer with exceptional photothermal and photodynamic effects, and it has been shown to be effective against various tumor cells. However, its low water solubility, poor stability, and limited tumor cell uptake have hindered its clinical application [21–23].

**Scheme 1:**
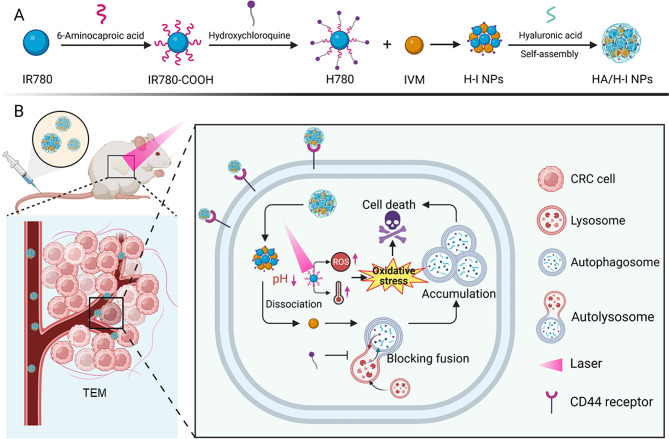
(**A**) Preparation diagram of HA/H-I NPs. (**B**) The underlying mechanism of HA/H-I NPs in CRC treatment. CRC, colorectal cancer; HA, hyaluronic acid; IVM, ivermectin; H-I NPs, H780-IVM nanoparticles; HA/H-I NPs, HA/H780-IVM nanoparticles; ROS, reactive oxygen species; TME, tumor microenvironment

In this study, we designed a photosensitizer H780 by conjugating IR780 and HCQ, which exhibits the ability to suppress autophagy effectively. Subsequently, a self-assembly method was employed to synthesize hyaluronic acid (HA) modified HA/H780-IVM NPs (HA/H-I NPs), with the aim of achieving synergistic photo-chemotherapy for colorectal cancer (Scheme 1). HA/H-I NPs could target CD44 to increase the uptake of HA/H-I NPs by tumor cells. Following internalization, HA/H-I NPs dissociated within the acidic tumor microenvironment (TME), resulting in the release of HCQ, IVM, and IR780 into the cytoplasm. Mechanistically, HCQ inhibits the protective autophagy induced by IVM, triggering apoptosis of CRC cells. Furthermore, IR780 generated hyperthermia and large amounts of reactive oxygen species (ROS) in tumor cells under NIR laser irradiation, further promoting CRC cell death in vivo and in vitro. In summary, our HA/H-I NPs possess significant potential as an innovative therapeutic system for the combined phototherapy and chemotherapy of colorectal cancer cells, which provides new insight into the synergistic treatment of colorectal cancer therapy.

## Materials and methods

### Materials

Ivermectin (I141334), IR780 (207399-07-3), 6-aminohexanoic acid (A306000), triethylamine (T103285), 4-dimethylaminopyridine (DMAP) (D109207), 1-ethyl-(3-dimethylaminopropyl) carbodiimide hydrochloride (EDCI) (E106172) and 1,3-Diphenylisobenzofuran (DPBF) were purchased from Aladdin Industrial Corporation. Hydroxychloroquine (BD134189) was purchased. Hyaluronic acid (MB7264) was purchased from Meilunbio. Dulbecco’s modified Eagle’s medium (DMEM), RPMI1640, was purchased from Thermo Fisher Science. Dimethyl sulfoxide (DMSO) crystal violet was purchased from Micros Sigma. Fetal bovine serum albumin (FBS) was provided by Biowest. Annexin V-FITC/PI cell apoptosis detection kit was purchased from Yeasen Biotechnology (Shanghai) Co., Ltd. LDH release detection kit was provided by Beyotime. 2’,7’-dichlorofluorescein diacetate (DCFH-DA) was purchased from Sigma-Aldrich.

### Synthesis of IR780-COOH

Briefly, IR780 (320 mg) was dissolved in 10 ml of anhydrous DMF, followed by the addition of 6-aminohexanoic acid (263.5 mg) and 278 µL of triethylamine. The reaction mixture was mixed under nitrogen protection at 85℃ under nitrogen protection at 85℃, avoiding light and stirring for 4 h. The solvent was removed by rotary evaporation and then purified on a silica gel column using methanol: dichloromethane (1:100, v/v) as the eluent to obtain IR780-COOH.

### Synthesis of H780

A mixture of IR780-COOH (100 mg), EDCI (40.6 mg), and NHS (123.0 mg) was stirred in anhydrous DMF (20 mL) under nitrogen protection at 0 °C for 30 min. After the reaction, HCQ (107.1 mg) and DMAP (26.0 mg) were added to the mixture after the solvent returned to room temperature. The reaction was stirred under nitrogen protection for 48 h, and the solvent was removed by rotary evaporation. The crude product was purified on silica gel column using methanol: dichloromethane (1:70, v/v) as the eluent to obtain H780.

### Preparation of HA/H-I NPs

In order to improve the stability of nanosystem, hyaluronic acid (HA) was used as a carrier to load H780 and IVM through self-assembly to prepare nanoparticles. Firstly, 7.5 mg of H780 and 6.0 mg of IVM were mixed with 1 mL of methanol solution and sonicated for 2 min. Then, the mixture was slowly added dropwise 10 mL of deionized water and stirred for 1 h. After that, the solvent in the methanol solution was evaporated by rotary evaporation at 35℃ for 10 min, resulting in a solution of H-I NPs. Subsequently, using ultrasonication, H-I NPs were slowly added to the HA solution and sonicated for 10 min to obtain the HA/H-I NPs solution.

### Characterization of HA/H-I NPs

The topography, particle size, and zeta potential of HA/H-I NPs were characterized using a JEM-200CX transmission electron microscope (TEM), a Brookhaven BI-200SM dynamic light scattering (DLS) instrument, and a Malvern laser particle size analyzer.

### Stability experiments of HA/H-I NPs

The prepared HA/H-I NPs and H-I NPs were stored in a refrigerator at 4 °C and the changes in their physical state were observed for 7 days, recorded and photographed. In addition, the prepared HA/H-I NPs solutions were diluted to appropriate concentrations and stored at 4 °C. The absorbance changes at the maximum absorption wavelength and the particle size changes of HA/H-I NPs were measured separately for 7 days.

### Single-linear state oxygen detection

1,3-Diphenylisobenzofuran (DPBF) was employed as a basis to detect the generation of reactive ROS under NIR laser irradiation of photosensitizers. We diluted IR780, H780, and HA/H-I NPs to 10 µM and added 30 µ L DPBF (dissolved in DMF, 1 mg·mL^− 1^) to 2 mL of water, IR780, H780, and HA/H-I NPs solutions, respectively. IR780 was irradiated with 808 nm NIR laser (1 W·cm^− 2^) for 5 min, and H780 and HA/H-I NPs were irradiated with 660 nm NIR laser (1 W·cm^− 2^) for 5 min. The absorbance of the solutions at 510 nm was recorded at 0 s, 30 s, 60 s, 90 s, 120 s, 180 s, 240 s, and 300 s after the NIR laser irradiation.

### In vitro release and encapsulation efficiency

This passage describes a method for releasing H780 and IVM from HA/H-I NPs using dialysis. The HA/H-I NPs solution is placed in various dialysis bags (MWCO = 3500 Da), with each bag containing 1 mL of solution. After sealing, all bags are immersed in 25 mL of phosphate buffer solution (PBS; pH 7.4 or 5.0) containing 5% (v/v) Tween-80 at 37 °C and shaken at a speed of 100 rpm. Then, 1 mL of the release medium is taken out and replaced with fresh release medium. The released amount of H780 is determined by UV-visible spectrophotometry (UV-8000 S), and the concentration is calculated based on the standard curve. The released amount of IVM is detected by high-performance liquid chromatography (Wooking HPLC K2025), and the concentration is calculated based on the standard curve. The encapsulation efficiency of HA/H-I NPs is also tested.

### Cell culture

RKO and HCT116 cells were cultured in DMEM medium containing 10% fetal bovine serum and 1% antibiotics, and CT26 cells were cultured in RPMI 1640 medium. These cells were maintained in a cell culture chamber at 37 °C in 5% humidified CO_2_.

### In vitro cell uptake

To perform cell uptake experiments, RKO and HCT116 cells were seeded onto 6-well plates at a density of 5 × 10^4^ cells per well and cultured for 1 day. After discarding the initial culture medium, fresh culture medium containing 10µM HA/H-I NPs was added into a 6-well plate at 0, 2, 4, 6, and 8 h, respectively. For qualitative analysis experiments, cells were washed three times with pre-chilled PBS, fixed with 4% paraformaldehyde for 15 min, and fluorescence pictures were taken by fluorescence inverted microscopy. For quantitative experiments, cells were washed 3 times with pre-chilled PBS and collected after trypsin digestion. 0 h group was used as a blank control and the cell fluorescence signal was recorded using a flow cytometer (BD Facsaria III).

### In vitro cell viability assay

RKO and HCT116 cells were seeded onto 96-well plates at a density of 3 × 10^4^ cells per well and cultured for 1 day. Then, the original medium was replaced with 200 µL medium that contained different concentrations of IVM, HCQ, IR780, H780, or HA/H-I NPs, and further cultured for 6 h. The cells were rinsed with pre-chilled PBS three times, and fresh medium was added. IR780 was irradiated with 808 nm laser for 3 min (1 W·cm^− 2^). H780 and HA/H-I NPs were irradiated with 660 nm laser for 3 min (1 W·cm^− 2^). After further incubation for 24 h, cell viability was detected by standard MTT assays. The cell survival rate was calculated according to the following equation:

$$\text{Cell}\,\text{Survival}\,\text{Rate}\,(\%) = \frac{As-Ab}{Ac-Ab}  \times 100\% $$

Where *As* is the absorbance of cells after treatment with medium of different samples plus drugs, *Ac* is the absorbance of the cells in the standard medium and *Ab* is the absorbance of the blank.

### Intracellular ROS assay

RKO and HCT116 cells were seeded onto 6-well plates at a density of 5 × 10^4^ cells per well and cultured for 1 day. When the attached cells reached a density of approximately 80%, the original medium was replaced with fresh medium containing PBS, IVM, H780, and HA/H-I NPs at a concentration of 10 µM and further cultured for 6 h. This was followed by three washes with prechilled PBS. Then, H780 and HA/H-I NPs were irradiated with 660 nm laser (1 W·cm^− 2^) for 3 min and incubated with serum-free culture containing DCFH-DA for 1 h. For qualitative analysis experiments, cells were washed 3 times with pre-chilled PBS, fixed in 4% paraformaldehyde for 15 min, and fluorescence photographs were taken by fluorescence inverted microscopy. For quantitative experiments, cells were washed 3 times with pre-chilled PBS and collected after trypsin digestion. The 0 h group served as a blank control, and the intracellular fluorescence signal was recorded by flow cytometry (BD Facsaria III).

### Clone formation analysis

Clone formation assays are used to assess the long-term effects of cell proliferation. RKO and HCT116 cells were seeded onto 12-well plates at a density of 5 × 10^3^ cells per well and cultured for 2 days. Then, cells were treated with the same concentrations of PBS, IVM, H780 and HA/H-I NPs for 6 h. After 3 washes with precooled PBS, H780 and HA/H-I NPs were irradiated with 660 nm laser (1 W·cm^-2^) for 3 min, and incubation was continued for 7 days. Then, they were fixed with 4% paraformaldehyde for 30 min and stained with crystalline violet overnight. A digital camera was used to take pictures and record.

### LDH analysis

RKO and HCT116 cells were seeded onto 96-well plates at a density of 5 × 10^4^ cells per well and cultured for 2 days. Then, the original medium was replaced with 200 µL medium containing different concentrations of IVM, H780 or HA/H-I NPs and further incubated for 6 h. The cells were rinsed three times with pre-chilled PBS, and medium without serum and double antibodies was added. H780 and HA/H-I NPs were irradiated with 660 nm laser for 3 min (1 W·cm^− 2^). Next, the cells were cultured overnight. Subsequently, the 96-well cell culture plates were placed in a multi-well centrifuge for 5 min at 400 g, and 120 µL of supernatant was carefully transferred from each well to a new 96-well cell culture plate using a pipette. Then, 60 µL of LDH working solution was added to each well of the new 96-well cell culture plate and incubated for 30 min at room temperature in a shaker using tin foil protected from light. Finally, the absorbance at 490 nm was measured using an enzyme marker to assess the effect of different drug treatment conditions on the cells.

### Apoptosis assay

The Annexin V-FITC Apoptosis Detection Kit was utilized to analyze the percentage of cells undergoing apoptosis. Briefly, cells were seeded into 6-well plates at a density of 5 × 10^4^ cells per well and cultured for 1 day. Cells were treated with the indicated concentrations of IVM, H780 and HA/H-I NPs for 6 h. After 3 washes with precooled PBS, H780 and HA/H-I NPs were irradiated with 660 nm laser (1 W·cm^− 2^) for 3 min, incubation was continued for 1 day. Then, cells was stained with Annexin V-FITC and PI combination for 15 min. Apoptosis was detected by flow cytometry according to the manufacturer’s protocol.

### Immunoblotting assay

Briefly, cells were inoculated into 6-well plates at a density of 5 × 10^4^ cells per well and incubated overnight. When the attached cells reached 75%, the original medium was replaced with fresh medium containing PBS, IVM, H780, and HA/H-I NPs at a concentration of 10 µM and further incubated for 6 h. Subsequently, the medium was washed three times with pre-cooled PBS and replaced with a fresh complete medium. Then, H780 and HA/H-I NPs were irradiated either with or without the 660 nm laser (1 W·cm^-2^) for 3 min, respectively, and the cells were incubated for 24 h. Cells were then collected, washed with pre-chilled PBS, and then lysed with radioimmunoprecipitation assay (RIPA) buffer (1% sodium deoxycholate, 1% Triton X-100, 10% SDS, supplemented with phosphatase inhibitors and protease inhibitors). Total lysates were subjected to sodium dodecyl sulfate-polyacrylamide gel electrophoresis (SDS-PAGE) and then transferred to polyvinylidene difluoride membranes and blocked with skim milk for 2 h at room temperature. Secondary antibodies were applied for 2 h after gentle overnight shaking at 4 °C with the indicated primary antibodies. Protein expression was detected by chemiluminescence.

### Animal model

Healthy BALB/c mice aged 6–8 weeks were selected as experimental subjects, and all in vivo experiments were performed by the standards of the Chengdu University Animal Use and Care System. Before completing the experimental manipulations, the mice were placed in a suitable environment for 2 weeks to help them adapt to their new environment. Then, we collected CT26 cells at the logarithmic growth stage and injected approximately 5 × 10^6^ CT26 cells into the right hind limb of each mouse by subcutaneous injection, thus establishing a tumor-bearing mouse model. Throughout the process, we strictly controlled the experimental conditions to ensure the safety and welfare of the mice (SYXK, sichuan, 2018 − 185; 2,022,312).

### In vivo imaging

When the tumor volume approached 100 mm^3^, 100 µL of H780 and HA/H-I NPs were slowly injected into the mice through the tail vein, respectively. The dose was equivalent to 3 mg/kg of H780 component. Images were taken at 1, 2, 4, 6, 8, and 24 h after injection using the in vivo imaging system (Xenogen IVIS Kinetic system). The BALB/c mice were sacrificed at 24 h after injection. Then the organs including the heart, liver, spleen, lung, kidney, brain, and tumor were collected for imaging biodistribution analysis by the imaging system.

### In vivo infrared imaging

When the tumor volume approached 100 mm^3^, 100 µL of saline, H780, and HA/H-I NPs were administered intravenously at a dose equivalent to 3 mg/kg of H780. After 4 h, the tumors of H780 and HA/H-I NPs treated mice were directly irradiated with 660 nm NIR laser. The temperature of the tumor site in mice was photographed and recorded using the NIR photothermal therapy imaging camera at 1-min intervals for a total of 5 min during NIR laser irradiation.

### Evaluation of in vivo anti-tumor effect

The tumor-bearing mice were randomly divided into 6 groups (*n* = 5 per group) receiving either physiological saline, IVM, H780, or HA/H-I NPs. Each group of mice received an equal dose of 3 mg/kg IVM, H780, or HA/H-I NPs via tail vein injection every 2 days. Four h after administration, the mice were subjected to direct irradiation at the tumor site with a wavelength of 660 nm for 3 min. During the treatment period, tumor volume and mouse weight were measured every two days.

The tumor volume was calculated as follows:

$$\text{T}\text{u}\text{m}\text{o}\text{r} \, \text{v}\text{o}\text{l}\text{u}\text{m}\text{e}=\frac{a{b}^{2}}{2}$$

Where the longest and shortest tumor diameters are *a* and *b*, respectively.

On the 15th day, the mice were anesthetized and the tumors and organs were removed. Finally, the tumor tissue was photographed and weighed.

The tumor suppression rate was calculated according to the following equation:

$$\text{T}\text{u}\text{m}\text{o}\text{r} \, \text{s}\text{u}\text{p}\text{p}\text{r}\text{e}\text{s}\text{s}\text{i}\text{o}\text{n} \, \text{r}\text{a}\text{t}\text{e}=\frac{Wc-Wt}{Wt}\times 100\%$$

Where *Wc* is the mean tumor weight in the NS group and *Wt* is the mean tumor weight in the treatment group.

### Histological examination

At the end of treatment, the relevant organs and tumors were separated and incubated in 4% paraformaldehyde solution. The associated tissues were embedded in paraffin, sectioned onto slides, and stained with hematoxylin and eosin (H&E). Associated tumors will also be frozen sectioned and fixed onto slides for further LC3B staining. Finally, tissue sections were observed with a light microscope (Nikon Eclipse Ci) and photographed.

### Statistical analyses

Statistical analysis was conducted using GraphPad Prism 8.0 software, and all data are presented as means ± standard error of the mean (SEM), unless otherwise specified. To compare different groups, Student’s t-test, ANOVA, or non-parametric ANOVA was used as appropriate and as indicated in the figure legends (*P < 0.05, **P < 0.01, ***P < 0.001).

## Results and Discussions

### Preparation and characterization of H780

**Fig. 1:**
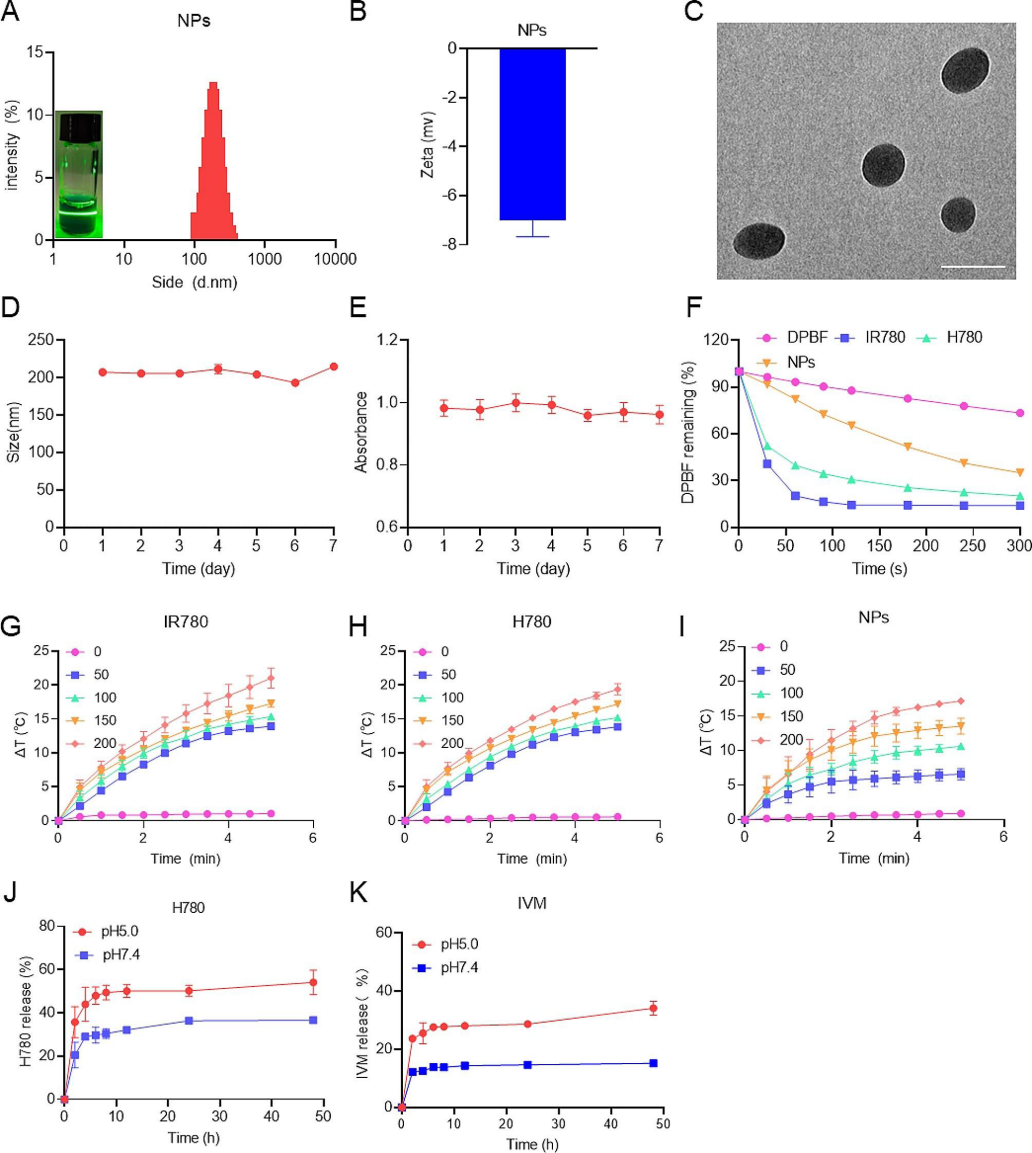
Characterization of HA/H-I NPs (NPs). (**A**) Size distribution of HA/H-I NPs and Tyndall effect of HA/H-I NPs. (**B**) Zeta potential of HA/H-I NPs. (**C**) TEM image of HA/H-I NPs; scale bar: 200 nm. (**D**) Changes in size absorbance of HA/H-I NPs over a week. (**E**) Changes in UV-vis absorbance of HA/H-I NPs over a week. (**F**) Residual levels of DPBF in different samples after NIR laser irradiation. (**G**) Temperature changes of IR780 with different concentrations upon 808 nm laser irradiation. (**H**) Temperature changes of H780 with different concentrations upon 660 nm laser irradiation. (**I**) Temperature changes of HA/H-I NPs with different concentrations upon 660 nm laser irradiation. (**J**) Release of H780 at pH 5.0 and pH 7.4. (**K**) Release of IVM at pH 5.0 and pH 7.4

We synthesized H780 with the autophagy inhibitor HCQ and IR780, and the related synthetic route is shown in Figure S1 (Supporting Information). Briefly, IR780 is modified by couplings with 6-aminohexanoic acid to form IR780-COOH. Then, HCQ was reacted with IR780-COOH to obtain HCQ-modified IR780, which is H780. The characteristic proton signals of IR780-COOH and H780 were shown in the ^1^H NMR spectra (Figure S2A-B), indicating the successful synthesis of IR780-COOH and H780. Subsequently, HA/H-I NPs were synthesized through self-assembly and characterized with a series of detection. We found that HA/H-I NPs and H-I NPs could be dispersed in the aqueous solution and exhibited the Tyndall effect (Figure S3A). The size of H-I NPs determined by the DLS method was 200 nm, and a positive potential of 3.47 mV (Figure S3B-C). TEM results indicate that the size distribution of H-I nanoparticles is not uniform (Figure S3D). The average diameter and zeta potential of HA/H-I NPs were 200 nm and − 7.02 mV, respectively (Fig. 1A-B), demonstrating the successful HA modification. In addition, nanoscale particle size (< 220 nm) can achieve the EPR effect, and negative potential can increase the stability of nanoparticles [24–27]. TEM analysis showed that the HA/H-I NPs were dispersed spherical particles, the size of which was consistent with the DLS result (Fig. 1C).

To investigate the stability of HA/H-I NPs, we examined their size distribution and maximum UV-vis wavelength for 7 days (Fig. 1D and E). We found no significant change in either of these parameters, and the solution did not show any precipitation (Figure S3E), suggesting that HA/H-I NPs were stable over this period. We found that the maximum UV-vis absorption wavelength of IR780 is 780 nm, while that of H780 is 638 nm (Figure S4A). The modification of IR780 with hydroxychloroquine altered its chemical structure and conjugated system, resulting in a shift of H780’s maximum absorption wavelength in the UV-vis spectrum. The H780 exhibited strong absorption in the NIR region, suggesting their potential suitability for phototherapy. When HA modifies H-I NPs, the maximum peak of the UV-vis absorption spectrum shifts to the right, which suggests that HA is successfully modified and implies that HA/H-I NPs might actively target tumor cells by recognizing CD44 receptors (Figure S4B). To verify the in vitro photosensitive properties of HA/H-I NPs, we used the DPBF probe to measure their ROS generation capability. As shown in Fig. 1F, we observed that the capability of HA/H-I NPs to generate ROS was inferior to that of IR780 and H780. However, it should be noted that after 5 min of laser irradiation, the efficiency of IR780 and H780 in generating ROS to reach a plateau, while HA/H-I NPs showed a continued tendency to produce ROS, suggesting that HA/H-I NPs could act as potential phototherapy agents for efficient ROS generation under prolonged laser exposure conditions. The results of in vitro temperature elevation also showed that HA/H-I NPs were able to raise the temperature by 16 ℃ under 660 nm laser irradiation, suggesting their ability for PTT (Fig. 1G-I).

In order to further clarify the drug content of HA/H-I NPs, we constructed a standard curve for H780 and IVM (Figure S5A, B). The experimental results indicate that H780 and IVM exhibit pH-responsive characteristics in HA/H-I NPs. Specifically, H780 can release approximately 44% under acidic conditions (Fig. 1J), while IVM can release approximately 34% under acidic conditions (Fig. 1K). These findings suggest that HA/H-I NPs can dissociate in the acidic tumor microenvironment, thereby reducing damage to normal cells. The IVM and H780 encapsulation efficiency of HA/H-I NPs were calculated at 80.87% and 83.75%, respectively.

### The cellular uptake and cytotoxicity of HA/H-I NPs

**Fig. 2:**
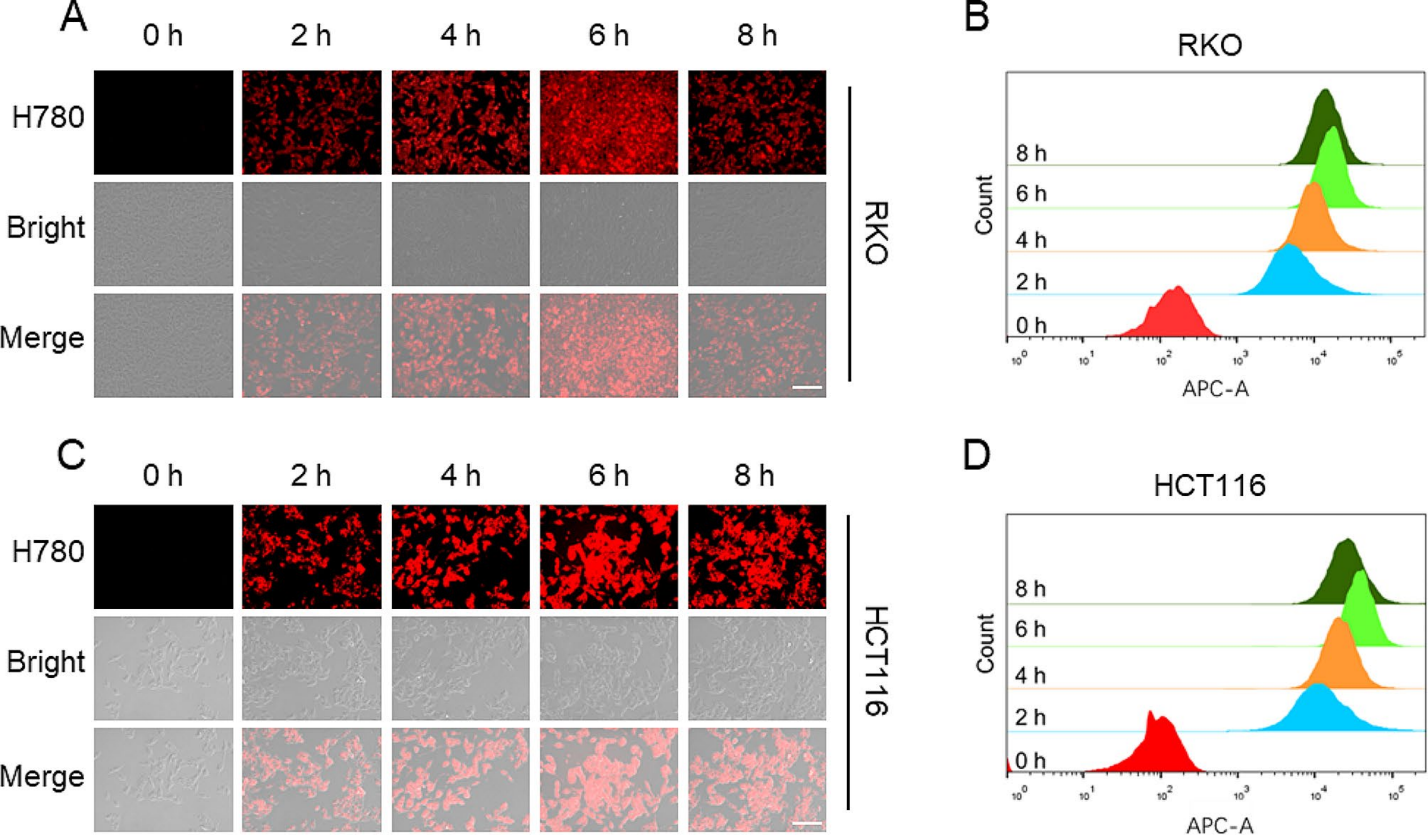
Intercellular uptake of HA/H-I NPs. (**A**) Fluorescence microscopy images showing cellular uptake of HA/H-I NPs in RKO cells; scale bar: 50 μm. (**B**) Flow cytometry showing cellular uptake of HA/H-I NPs in RKO cells. (**C**) Fluorescence microscopy images showing cellular uptake of HA/H-I NPs in HCT116 cells; scale bar: 50 μm. (**D**) Flow cytometry showing cellular uptake of HA/H-I NPs in HCT116 cells

The effective uptake of drugs by tumor cells is a crucial step that affects the outcome of cancer treatment [28]. We used fluorescence microscopy and flow cytometry to investigate the uptake behavior of colon cancer cells toward HA/H-I NPs. As shown in Fig. 2, the results from fluorescence microscopy indicated that colon cancer cells could reach the maximum uptake of drugs at 6 h, consistent with the results from flow cytometry. However, at 8 h, the fluorescence of drug uptake showed a downregulation, suggesting that 6 h could be the optimal time for drug intervention to treat tumors. Consequently, we conducted subsequent in vitro experiments involving infrared laser irradiation, following a 6-hour culture period with HA/H-I NPs.

### In vitro cytotoxicity study of HA/H-I NPs

To evaluate the in vitro cytotoxicity of HA/H-I NPs against tumor cells, we conducted a series of experiments. These experiments included: using the MTT assay to measure the metabolic status of the cells; employing the LDH release assay to evaluate the damage of HA/H-I NPs to cell membrane integrity; utilizing the colony formation assay to observe the effect of HA/H-I NPs on cell proliferation; and conducting apoptosis detection experiments to study the ability of HA/H-I NPs to promote cell apoptosis.

**Fig. 3:**
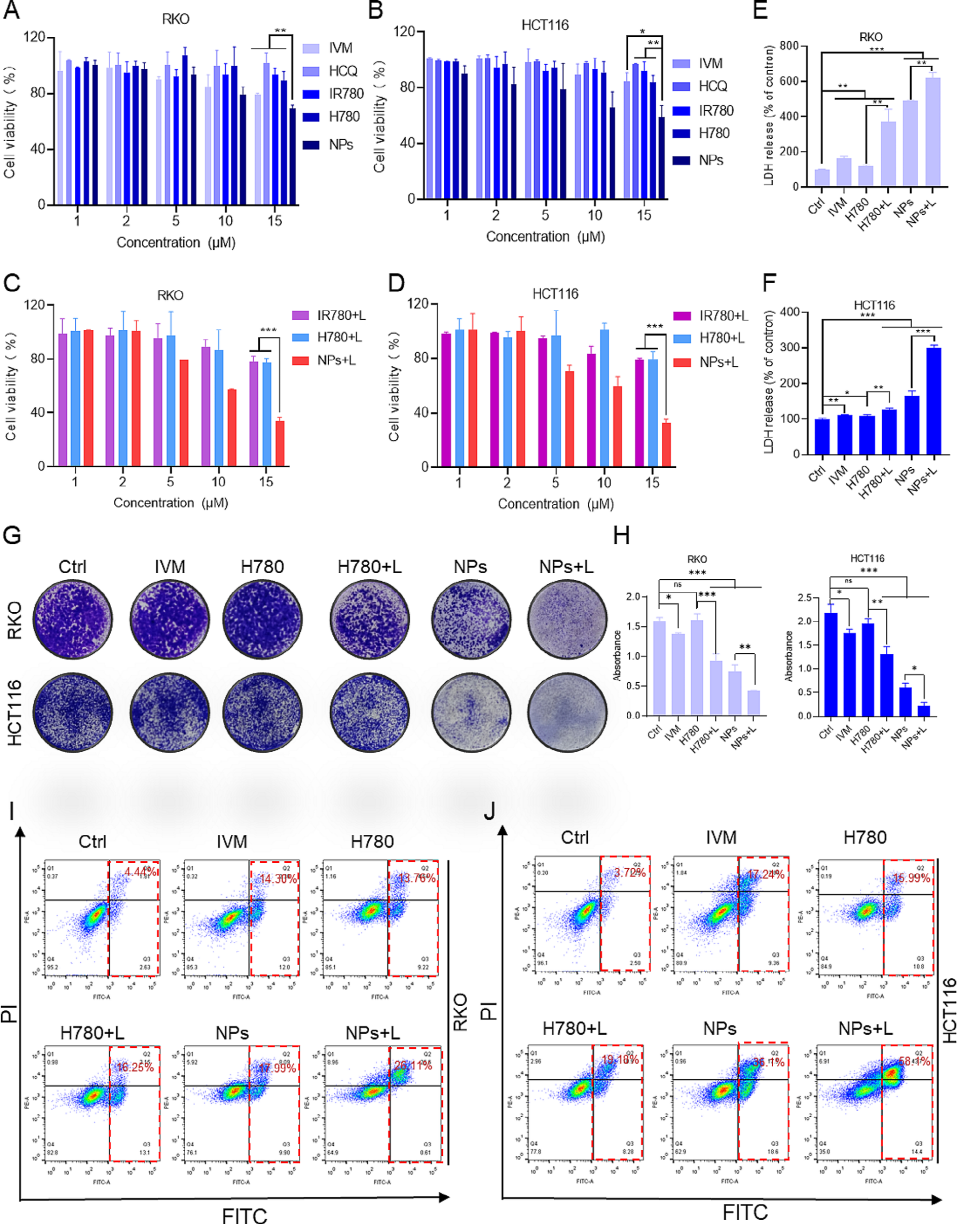
The cytotoxicity of HA/H-I NPs in vitro. (**A**-**B**) Cell viability of RKO (**A**) and HCT116 (**B**) cells co-cultured with free IVM, HCQ, IR780, H780, and HA/H-I NPs (*n* = 3). (**C**-**D**) Cell viability of RKO (**C**) and HCT116 (**D**) cells co-cultured with free IR780, H780, and HA/H-I NPs with NIR laser irradiation (*n* = 3). (**E**-**F**) Analysis of LDH release in the co-cultured RKO (**E**) and HCT116 (**F**) cells incubated with different treatments (*n* = 3). (**G**) Representative images of colony formation of RKO and HCT116 cells after different treatments. (**H**) Statistical graph of colony formation of RKO and HCT116 cells after different treatments. (*n* = 3). (**I**-**J**) Flow cytometry results of apoptosis in RKO (**I**) and HCT116 (**J**) cells under different treatments. Data are shown as means ± SD (*n* = 3, one-way ANOVA). ^*^P < 0.05, ^**^P < 0.01, ^***^P < 0.001, ns, not significant

Results from the MTT assay showed that the use of HCQ, IR780, and H780 alone had almost no significant impact on the vitality of tumor cells. In contrast, IVM and HA/H-I NPs exhibited a slight inhibitory effect on cells (Fig. 3A, B, and S6A). Following laser irradiation, HA/H-I NPs demonstrated significant antitumor effects, while IR780 and H780 showed phototherapeutic effects (Fig. 3C, D, and S6B), further confirming the effectiveness of HA/H-I NPs in killing colorectal cancer cells.The results of the LDH release assay indicate that treatment with HA/H-I NPs significantly increased LDH release, and this release was further enhanced following NIR irradiation. This suggests that HA/H-I NPs not only kill colorectal cancer cells but also compromise their cellular membrane integrity (Fig. 3E, F). The results of the colony formation assay showed a significant reduction in the number of cell colonies formed after treatment with HA/H-I NPs, indicating that these nanoparticles effectively inhibit the proliferation of colorectal cancer cells (see Fig. 3G, H). Furthermore, the measurement of cell apoptosis using the Annexin V/PI double staining method further confirmed that HA/H-I NPs could promote the apoptosis of tumor cells (Fig. 3I, J). In summary, our research findings demonstrate that the HA/H-I NPs we developed can effectively inhibit the survival and proliferation of colorectal cancer cells and promote their death, exhibiting promising cytotoxic properties.

### HA/H-I NPs evoke apoptosis of CRC cells by manipulating autophagy

**Fig. 4:**
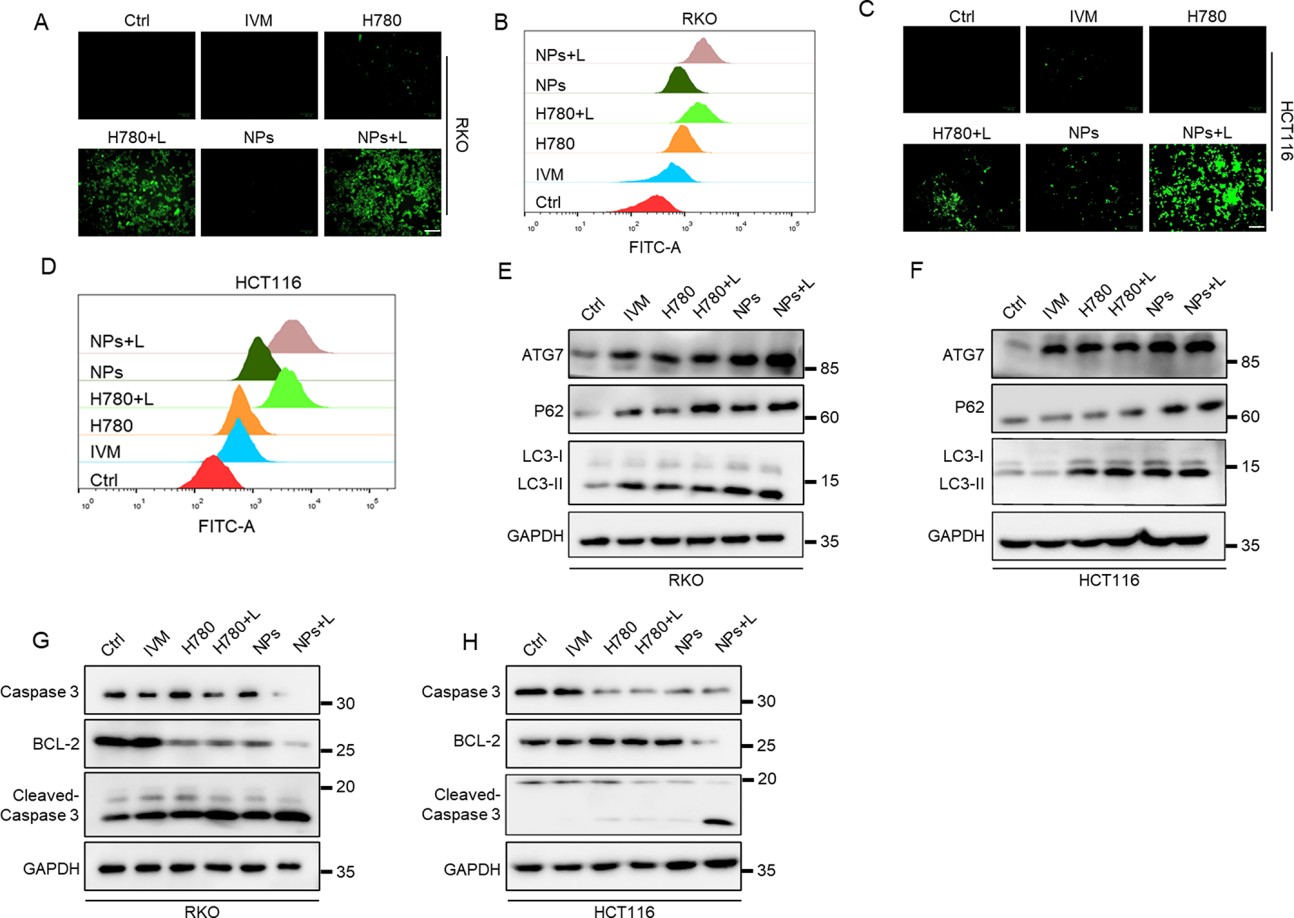
The mechanism of HA/H-I NPs inducing tumor cell apoptosis. (**A**) Fluorescence microscopy images analysis for intracellular ROS generation of RKO cells using DCFH-DA as a probe. Scale bar: 50 μm. (**B**) Flow cytometry analysis for intracellular ROS generation of RKO cells using DCFH-DA as a probe. (**C**) Fluorescence microscopy images analysis for intracellular ROS generation of HCT116 cells using DCFH-DA as a probe. Scale bar: 50 μm. (**D**) Flow cytometry analysis for intracellular ROS generation of HCT116 cells using DCFH-DA as a probe. (**E**) Immunoblot analysis of autophagy markers for RKO cells treated with different treatments. (**F**) Immunoblot analysis of apoptotic markers for HCT116 cells treated with different treatments. (**G**) Immunoblot analysis of autophagy markers for RKO cells treated with different treatments. (**H**) Immunoblot analysis of apoptotic markers for RKO cells treated with different treatments

In order to elucidate the mechanism of HA/H-I NPs in inhibiting CRC cells, we used DCFH-DA probes to detect the levels of intracellular ROS after different interventions. The results from fluorescence microscopy and flow cytometry (Fig. 4A-D) showed that H780 produced a fluorescence signal after NIR laser irradiation, but HA/H-I NPs + Laser can significantly increase the fluorescence intensity in cancer cells, suggesting that light irradiation is able to enhance the level of oxidative stress induced by HA/H-I NPs, thus promoting the damage or death of tumor cells as an upstream molecular event. We also found that IVM can induce upregulation of ROS levels, but the fluorescence intensity is inconspicuous. These phenomena are possibly due to its low dosage and withdrawal after 6 h, leading to most drugs not entering the cancer cells and making it difficult to exert a therapeutic effects. H780 and HA/H-I NPs without light exposure did not show a significant increase in intracellular ROS levels, indicating that NIR light exposure is a critical factor in activating the therapeutic effect of HA/H-I NPs.

Studies have shown that ROS can be a critical triggering factor to activate cell autophagy [29, 30]. The level of cell autophagy and the smoothness of autophagic flow are important factors that affect cell fate [31]. Previous studies have shown that IVM can induce protective autophagy in cancer cells, thereby inhibiting its ability to induce cell apoptosis [8, 32]. After co-treatment with the autophagy inhibitor HCQ, the autophagic flow in tumor cells was blocked, thus promoting the apoptosis of tumor cells. Similarly, phototherapy can also induce protective autophagy in cells, weakening its inhibitory effect on tumor cells and leading to phototherapy tolerance [33, 34]. In our study, we observed a significant upregulation of the key autophagy proteins ATG7 and LC3II after different treatments, indicating the occurrence of autophagy in CRC cells. Furthermore, the expression of the autophagy substrate P62 was significantly increased following treatment with H780, H780 + L, HA/H-I NPs, and HA/H-I NPs + Laser, suggesting an interference with the formation of autophagolysosomes and resulting in the blockade of autophagic flux (Fig. 4E, F). Subsequently, we assessed the expression levels of key apoptosis proteins BCL-2, caspase-3, and cleaved caspase-3. The results were consistent with those of autophagy-related proteins. In the groups where P62 expression increased, there was a significant elevation in the level of apoptosis, as confirmed by flow cytometry analysis, indicating the occurrence of apoptosis in colorectal cancer cells (Fig. 4G, H). These results indicated that HA/H-I NPs could promote ROS production under light conditions, inducing protective autophagy in CRC cells. Interestingly, HCQ inhibited the occurrence of protective autophagy and blocked autophagic flow, thereby triggering CRC cell apoptosis. These findings also confirmed the intrinsic mechanism of HA/H-I NPs in causing tumor cell apoptosis through modulating autophagy.

**Fig. 5:**
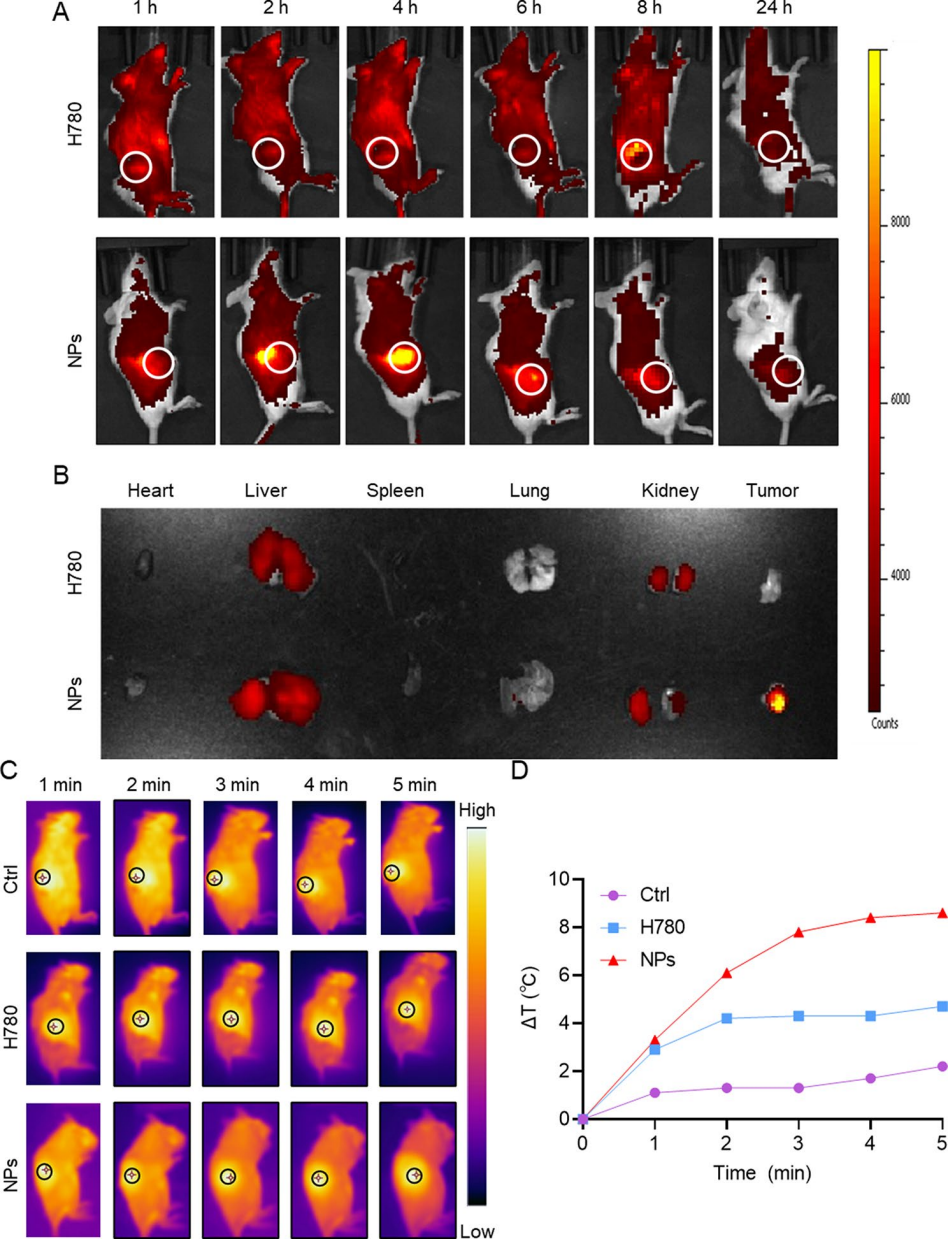
In vivo biological distribution and in vivo warming of HA/H-I NPs. (**A**) The biodistribution of H780 and HA/H-I NPs in tumor-bearing mice at various time points. (**B**) Fluorescence imaging of dissected organs and tumors at 24 h. (**C**) Infrared thermal images of tumor-bearing mice during laser irradiation at the tumor sites after intravenous injections of NS, free H780, and HA/H-I NPs. (**D**) temperature-change curves at the tumor sites after intravenous injections of NS, free H780, and HA/H-I NPs

The tumor-targeting ability of the nanoplatform is one of the most critical features of antitumor efficiency. Nanodrug targeting tumor cells can reduce the damage caused by chemotherapeutic drugs to normal tissues [35]. Therefore, we used NIRF imaging of photosensitizers to track the biodistribution of H780 and HA/H-I NPs at different time points in the tumor-bearing BALB/c mice model. As shown in Fig. 5A, the fluorescent signal of H780 was diffuse in vivo, with fluorescence aggregation occurring only at 8 h after injection. Nonetheless, real-time fluorescence imaging showed that HA/H-I NPs aggregated at the tumor site with the maximum fluorescence intensity 4 h after administration, demonstrating the active targeting property of HA/H-I NPs to CD44 receptors of the tumor. Therefore, NIR laser irradiation 4 h after tail vein administration was selected for subsequent experiments. Moreover, HA/H-I NPs exhibited prolonged fluorescence signals at 24 h after injection, indicating the slow-release ability for PDT and PTT. The major organs and the tumor tissue were then obtained to investigate the accumulation efficiency after 24 h. Unsurprisingly, the results showed that the fluorescence signal of HA/H-I NPs in tumor tissue was maintained after 24 h, while H780 was quenching, indicating the targeting ability of HA/H-I NPs in tumor therapy (Fig. 5B).

Hyperpyrexia can accelerate blood flow to the tumor site and improve the tumor tissue permeability, which is favorable for the uptake of NPs by tumor cells and the deep penetration of NPs into the tumor tissue [36, 37]. Then, we evaluated the PTT ability of HA/H-I NPs in vivo. As shown in NIR photothermal images and heating curves (Fig. 5C and D), after NIR laser irradiation, the temperature increased at the tumor site in the control group was negligible, and the H780 group also increased slightly. Notably, the surface temperature of HA/H-I NPs was elevated by nearly 10 °C and reached the efficiency of hyperpyrexia. These results showed the photothermal capability of HA/H-I NPs in vivo treatment of CRC.

### Anti-CRC effects of HA/H-I NPs in vivo

**Fig. 6:**
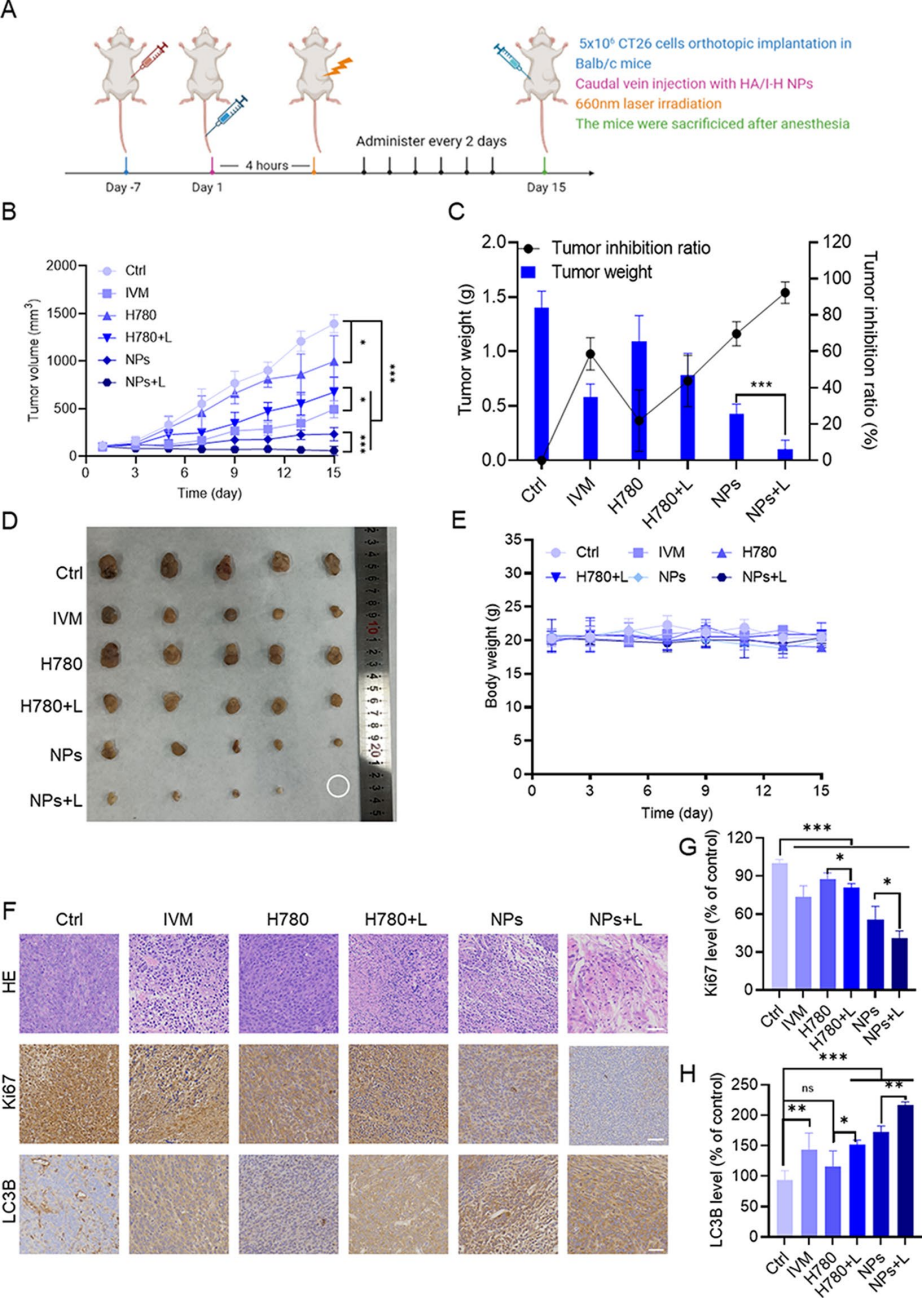
Anti-colorectal-cancer potential of HA/H-I NPs in vivo. (**A**) Schematic illustration of the anti-CRC potential of HA/H-I NPs in mice model (*n* = 5). (**B**) Tumor volume change curves of mice. (**C**) Average tumor weight and corresponding tumor inhibition ratios of mice. (**D**) Corresponding tumor photographs. (**E**) The body weight changes of mice. (**F**) H&E and Immunohistochemistry images of tumor slices. Scale bar: 50 μm. (**G**) Ki67 positive rate in tumor tissue. (**H**) LC3B positive rate in tumor tissue. Data are shown as means ± SD (*n* = 5, one-way ANOVA). ^*^P < 0.05, ^**^P < 0.01, ^***^P < 0.001, ns, not significant

Owing to the favorable tumor-targeting ability, we evaluated the photothermal potential of HA/H-I NPs in vivo. CT-26 tumor-bearing BALB/c mice models were established for further evaluation of the tumor inhibitory effect of HA/H-I NPs. After 7 days of CT-26 cells inoculation, mice were intravenously injected with saline, IVM, H780, and HA/H-I NPs every other day for 15 days, either with or without NIR irradiation (Fig. 6A). As shown in Fig. 6B-D, the analysis of tumor volume and weight revealed variations in the inhibitory effects on tumor tissues across different treatment methods. Notably, the treatment groups of HA/H-I NPs and HA/H-I NPs + Laser demonstrated significant antitumor efficacy, with tumor inhibition rates reaching 69.7% and 92.4%, respectively. It is particularly important to highlight that there was a significant difference in both tumor volume and weight between the HA/H-I NPs + Laser treatment group and the group treated only with HA/H-I NPs. These results suggested that HA/H-I NPs could satisfactorily suppress the growth of CRC cells in vivo under Laser irradiation. Notably, the body weight of mice in the HA/H-I NPs + Laser group did not change significantly during the treatment period (Fig. 6E), indicating the safety of the treatment regimen. Moreover, H&E staining and IHC staining of tumor tissues were examined to investigate the in-depth mechanism of HA/H-I NPs in CRC therapy. As shown in Fig. 5F, incomplete structure and reduced nuclei in H&E staining in the HA/H-I NPs + Laser group were observed, indicating the occurrence of apoptosis. Positive IHC staining of LC3B and attenuated Ki67 demonstrated the inhibition of autophagy and proliferation (Fig. 6F-H). Subsequently, we investigated the biosafety of HA/H-I NPs in vivo. H&E staining analysis of major organs and serum biochemical analysis showed that all treatment groups had negligible influence on the physiological function of mice, which verified the safety of HA/H-I NPs for CRC treatment in vivo (Figure S7A-E).

## Conclusions

In summary, we successfully constructed efficient and low-toxic photo-chemotherapy nanoplatform HA/H-I NPs, which could effectively inhibit autophagy and promote apoptosis in CRC cells. In this nanoplatform, the photosensitizer IR780 was first linked to HCQ to create H780, which subsequently self-assembled with IVM to form H-I NPs. Finally, HA was modified to synthesize HA/H-I NPs to target tumor cells. HA/H-I NPs were able to accumulate at tumor sites, and the released IVM could induce apoptosis in tumor cells, with H780 augmenting the cytotoxicity of IVM by blocking autophagy. In addition, H780 could generate hyperthermia and elevated levels of ROS at the tumor site to kill tumor cells via NIR laser irradiation. The results of in vitro and in vivo experiments revealed that HA/H-I NPs exhibited exceptional anti-CRC effects. Therefore, our study has provided a new strategy for the treatment of CRC that could enhance chemo-phototherapy by interrupting autophagy.

### Electronic supplementary material

Below is the link to the electronic supplementary material.

Supplementary Material 1

## Data Availability

All data generated or analyzed during this study are included in the article.
