# Supplementary material for: Drug repurposing-based nanoplatform via modulating autophagy to enhance chemo-phototherapy against colorectal cancer
Source: J Nanobiotechnology. 2024 Apr 24;22:202. doi: 10.1186/s12951-024-02416-5 (PMC11040740; doi:10.1186/s12951-024-02416-5)
Supplement: Supplementary file 1 — Supplementary Material 1 [file 12951_2024_2416_MOESM1_ESM.docx]

**Supporting Information**

**Drug Repurposing-based Nanoplatform Via Modulating Autophagy to Enhance Chemo-phototherapy Against Colorectal Cancer**

Ke Ding^a,b,1^, Hailong Tian^c,1^, Lei Li^d,1^, Zhihan Wang^c^, Shanshan Liu^b^, Ning Ding^e^, Edouard C. Nice^f^, Canhua Huang^c^, Jinku Bao^c*^, Wei Gao^a*^, Zheng Shi^a,b*^.

*^a^Clinical Medical College, Affiliated Hospital of Chengdu University, Chengdu University, Chengdu, 610106, China.*

*^b^Department of Clinical Pharmacy, School of Pharmacy, Zunyi Medical University, Zunyi, 563006, China.*

*^c^State Key Laboratory of Biotherapy and Cancer Center, West China Hospital, and West China School of Basic Medical Sciences & Forensic Medicine, Sichuan University, and Collaborative Innovation Center for Biotherapy, Chengdu, 610041, China.*

*^d^Department of anorectal surgery, Hospital of Chengdu University of Traditional Chinese Medicine and Chengdu University of Traditional Chinese Medicine, Chengdu, 610072, China.*

*^e^Shanghai municipal Hospital of Traditional Chinese Medicine, Shanghai University of Traditional Chinese Medicine, 201203, China.*

*^f^Department of Biochemistry and Molecular Biology, Monash University, Clayton, VIC, 3800, Australia.*

*^1^Authors contributed equally*

*^*^Corresponding authors.*


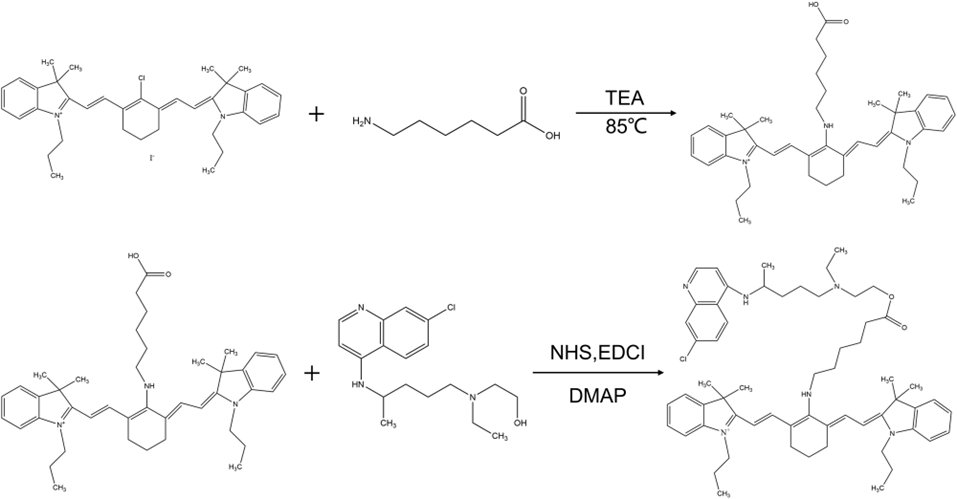


**Figure S1.** Synthetic route of H780.


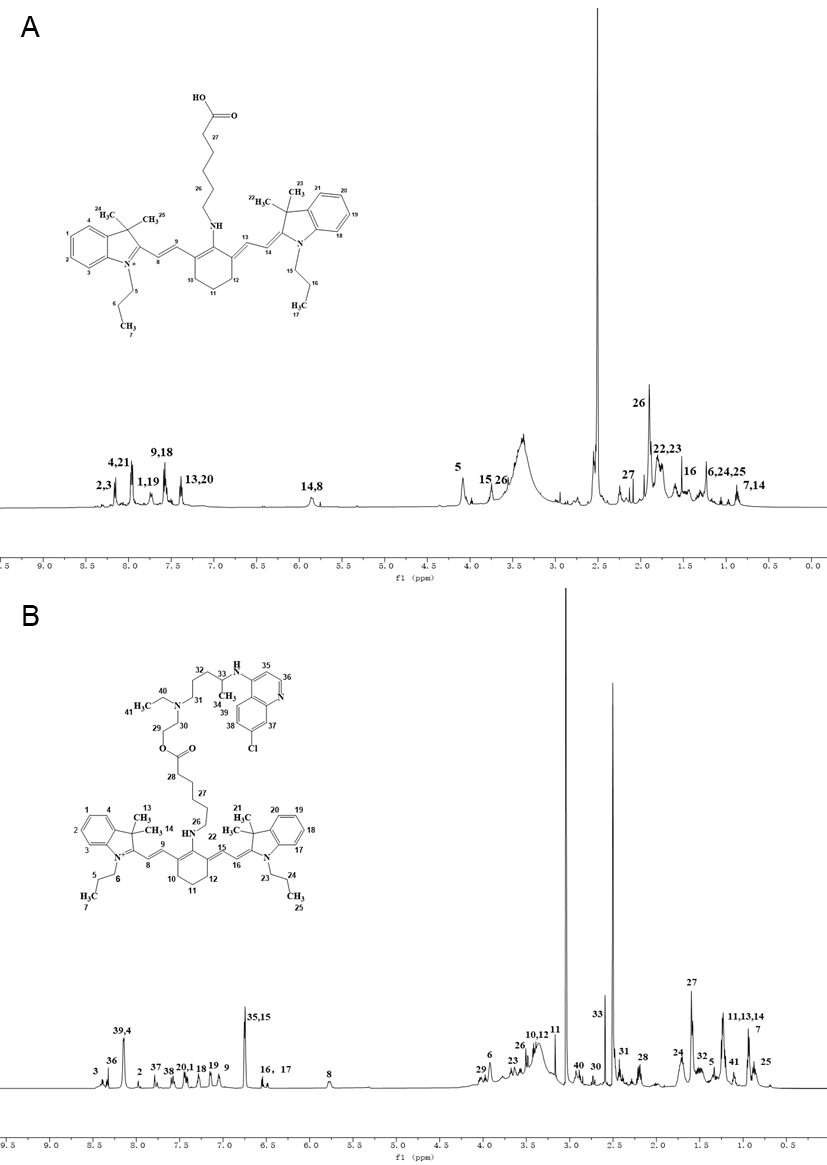


**Figure S2. ^1^H NMR characterization of compounds characterization of compounds.** (A) ^1^H NMR spectrum of IR780-COOH in DMSO-*d_6._* (B) ^1^H NMR spectrum of H780 in DMSO-*d_6_*_._


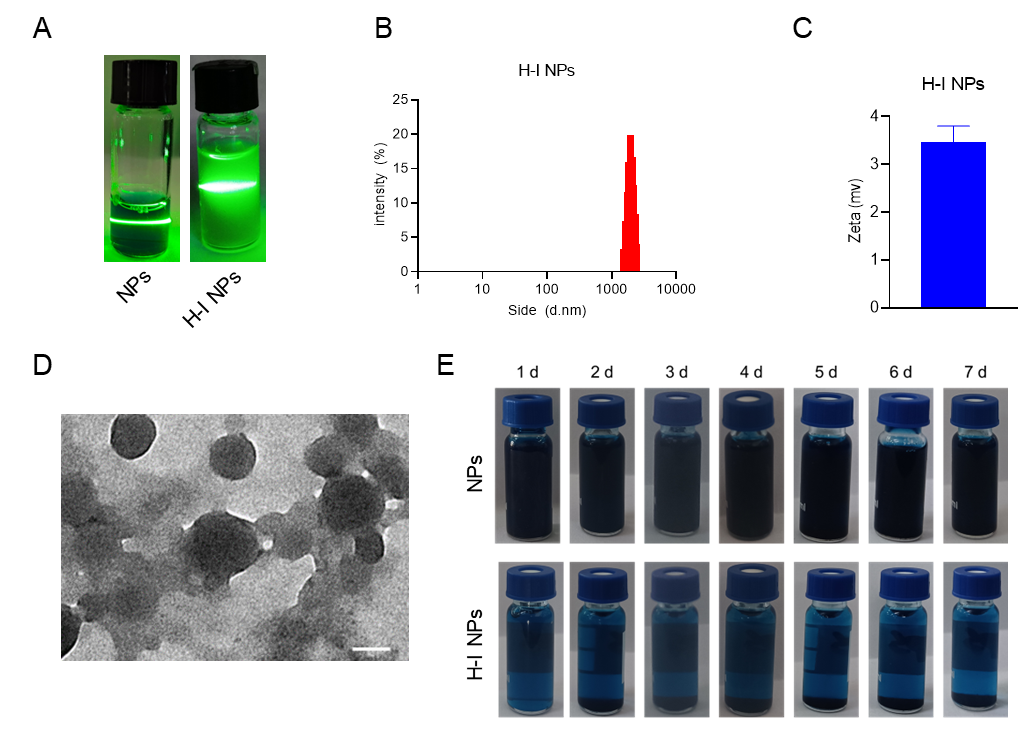


**Figure S3. Characterization of HA/H-I NPs and H-I NPs.** (A) Tyndall effect of HA/H-I NPs and H-I NPs. (B) Size distribution of H-I NPs. (C) Zeta potential of H-I NPs. (D) TEM image of H-I NPs; scale bar: 1000 nm. (G) Changes in HA/H-I NPs and H-I NPs within a week.


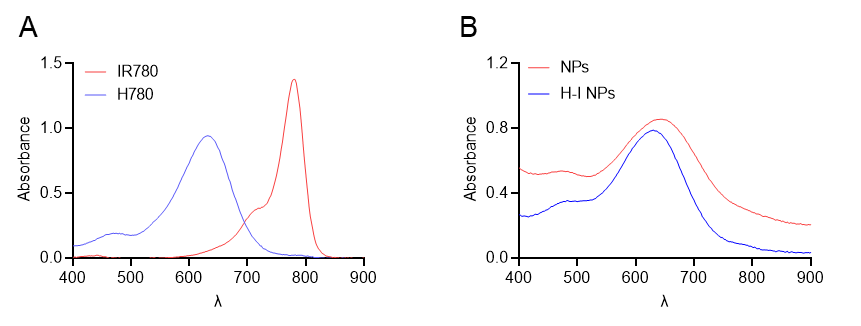


**Figure S4.** **UV-vis spectra.** (A) UV-vis spectra of IR780 and H780. (B) UV-vis spectra of HA/H-I NPs and H-I NPs.


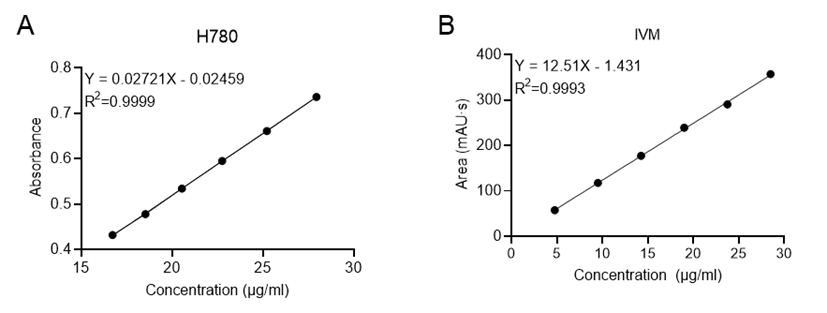


**Figure S5.** **standard curve.** (A) H780 of standard curve. (B) IVM of standard curve.


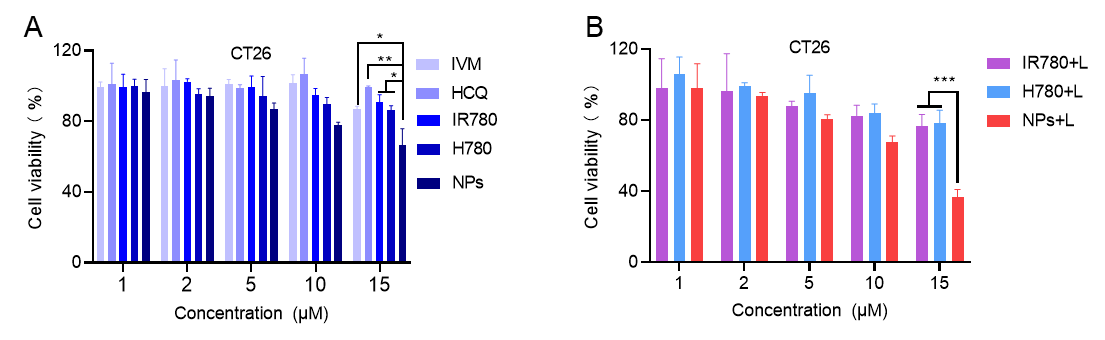


**Figure S6. The cytotoxicity of HA/H-I NPs *in vitro*.** (A) Cell viability of RKO co-cultured with free IVM, HCQ, IR780, H780 and HA/H-I NPs (n=3). (B) Cell viability of RKO co-cultured with free IR780, H780 and HA/H-I NPs with NIR laser irradiation (n=3). Data are shown as means ± SD (n=3, one-way ANOVA). *^*^P* < 0.05, *^**^P* < 0.01, *^***^P* < 0.001, ns, not significant.


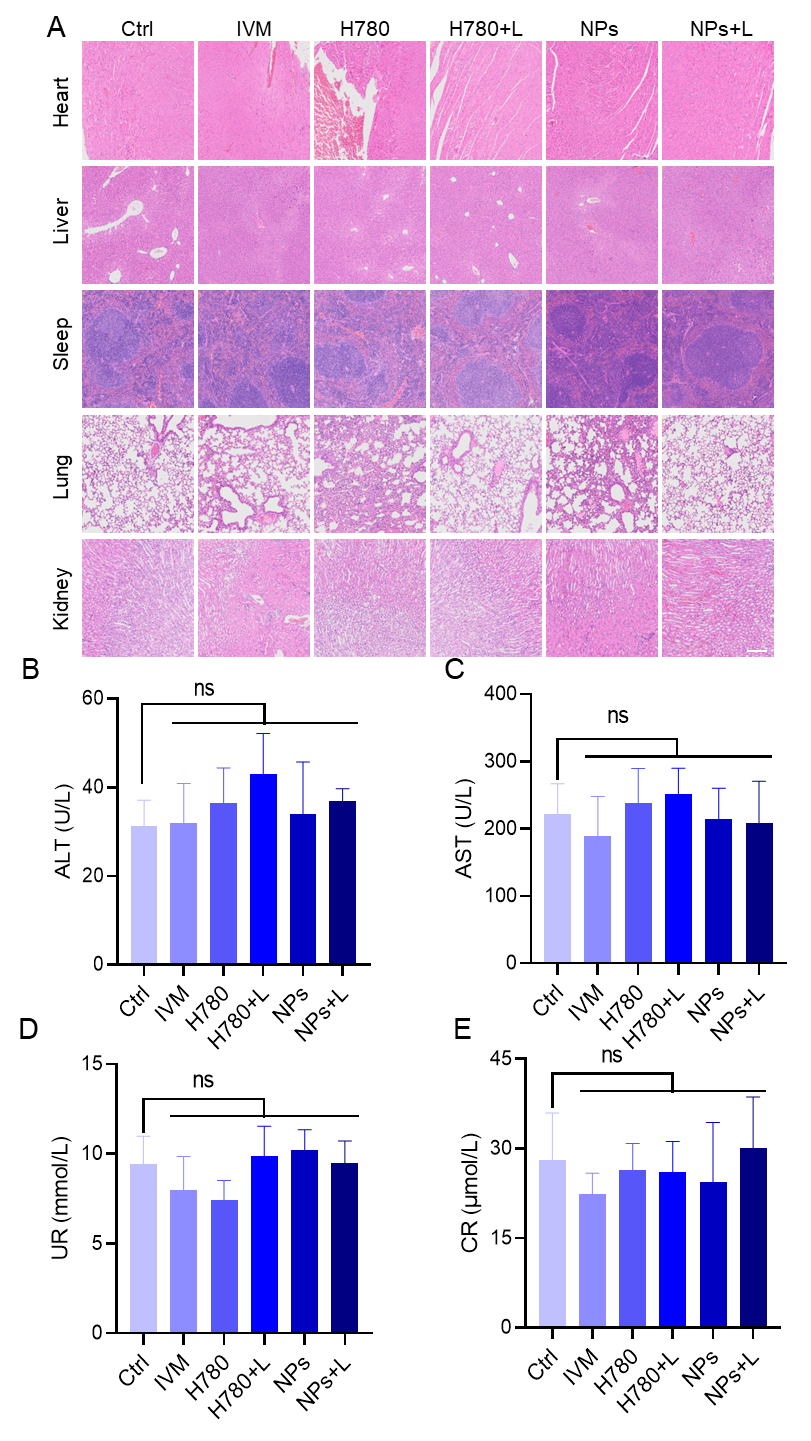


**Figure S7. *In vivo* biosafety of HA/H-I NPs.** (A) H&E stained images of major organs (heart, liver, spleen, lung and kidney) after treating various formulations. Scale bar: 100 μm. (B) Blood biochemical markers of ALT. (C) Blood biochemical markers of AST. (D) Blood biochemical markers of UR. (E) Blood biochemical markers of CR (n=3). Data are shown as means ± SD (n=3, one-way ANOVA). *^*^P* < 0.05, *^**^P* < 0.01, *^***^P* < 0.001, ns, not significant.
